# Thyroid Function Testing in Hospitalized Patients: A Practical Framework for Clinicians

**DOI:** 10.7759/cureus.106544

**Published:** 2026-04-06

**Authors:** Nidha Shapoo, Noella Boma

**Affiliations:** 1 Internal Medicine, New York Medical College, Metropolitan Hospital Center, New York, USA; 2 Internal Medicine, Metropolitan Hospital, New York, USA

**Keywords:** diagnostic algorithm, hospitalized patients, non-thyroidal illness syndrome, thyroid function tests, thyroid-stimulating hormone

## Abstract

Thyroid-stimulating hormone (TSH) is the primary screening test for thyroid issues in outpatient settings; however, interpreting it in hospitalized patients is more complex. Acute illness, medication effects, and assay interference can significantly influence thyroid function tests, often without underlying thyroid disease. Non-thyroidal illness syndrome, also called euthyroid sick syndrome, is common in hospitalized and critically ill patients and frequently causes temporary changes in thyroid hormone levels that can mimic primary thyroid disorders. Understanding when to test thyroid function in hospitalized patients is crucial for proper interpretation and clinical decision-making.

This narrative review examines the physiology of thyroid hormone action and how the hypothalamic-pituitary-thyroid axis changes in the hospital setting. We also summarize the clinical presentation and management of thyroid emergencies and highlight situations in which TSH testing is most useful. Additionally, we provide practical guidance on interpreting thyroid function tests in hospitalized patients and identify key clinical scenarios that require endocrinology consultation. Finally, we propose a practical diagnostic algorithm to help residents and medical students distinguish transient thyroid function abnormalities caused by acute illness and to guide appropriate evaluation and management in the inpatient setting.

## Introduction and background

Thyroid disease is common in ambulatory medicine, affecting an estimated 4-10% of the adult population. Thyroid-stimulating hormone (TSH) is the initial screening test for thyroid abnormalities in the outpatient setting. If TSH is outside the reference range, free T4 is measured. Overt primary hypothyroidism is diagnosed when the TSH is elevated and the FT4 is low. A decision to treat is often made when TSH is >10 mU/L, even if the FT4 is within the reference range. A high TSH level (usually less than 10 mU/L) in the presence of normal FT4 and FT3 is most often due to subclinical thyroid dysfunction. Hyperthyroidism is diagnosed when TSH is low, and FT4 is elevated [1,2].

The inpatient environment presents a fundamentally different physiological landscape. Acute systemic illness, critical care interventions, and pharmacologic exposures alter thyroid function tests in complex and often adaptive ways. Routine testing for TSH is not recommended for hospitalized patients admitted with acute illness unless hyperthyroidism or hypothyroidism is suspected, as acute illness can transiently alter the hypothalamic-pituitary-thyroid axis, leading to misleading results, unnecessary investigations, and potential overtreatment. Studies suggest that a substantial proportion of thyroid function tests ordered in hospitalized patients are performed without a clear clinical indication, with estimates ranging from approximately 20% to 40%, contributing to diagnostic uncertainty and potential overtreatment [3-7]. Understanding when thyroid testing adds value and when it does not is central to high-quality inpatient care. The following clinical scenario illustrates this challenge:

A 72-year-old man with a history of coronary artery disease and chronic kidney disease was admitted for acute decompensated heart failure. During hospitalization, thyroid function tests were obtained despite the absence of symptoms suggestive of thyroid dysfunction. Laboratory results revealed an elevated TSH level of 7.5 mIU/L with a normal free thyroxine level. This was interpreted as subclinical hypothyroidism, and levothyroxine therapy was initiated during the hospital stay. The patient was discharged on thyroid hormone replacement without a clear plan for reassessment. At follow-up six weeks later, the patient reported new-onset palpitations and anxiety. Repeat testing demonstrated a suppressed TSH level, consistent with iatrogenic thyrotoxicosis. Levothyroxine was subsequently discontinued, and thyroid function normalized on repeat evaluation.

In retrospect, the initial TSH elevation was likely transient and related to acute illness and recovery, consistent with non-thyroidal illness syndrome. This case highlights how inappropriate inpatient thyroid testing and premature treatment of mild abnormalities can lead to avoidable harm, particularly in patients with underlying cardiovascular disease. This review aims to provide internists with a structured, practical approach to thyroid hormone testing in hospitalized adults.

## Review

Methods

This narrative review was conducted using a structured literature search of PubMed and Google Scholar databases to identify relevant studies on thyroid function testing in hospitalized patients. Search terms included “thyroid-stimulating hormone,” “non-thyroidal illness syndrome,” “euthyroid sick syndrome,” “inpatient thyroid testing,” and “thyroid dysfunction in acute illness.” Priority was given to guideline-based recommendations, including those from the American Thyroid Association (ATA) and American Association of Clinical Endocrinology (AACE), as well as clinically relevant observational studies and review articles. Reference lists of selected articles were also reviewed to identify additional relevant sources. Articles were selected based on relevance to inpatient thyroid function testing and clinical applicability to internal medicine practice.

This review was conducted as a qualitative narrative synthesis of the available literature. No formal statistical analysis or quantitative pooling of data (e.g., meta-analysis or meta-regression) was performed. Evidence was synthesized descriptively, with emphasis on clinical applicability, guideline-based recommendations, and consistency across studies.

Physiology and function of thyroid hormones

TSH, also called thyrotropin, is a glycoprotein produced by the anterior pituitary and regulated by the hypothalamic-pituitary-thyroid (HPT) axis. The HPT axis is a vital neuroendocrine system regulating metabolism, growth, and development via a negative feedback loop. The hypothalamus releases thyrotropin-releasing hormone (TRH), which binds to TRH receptors on thyrotropic cell membranes in the anterior pituitary, thereby stimulating the release of TSH. The primary target of TSH is the thyroid gland. TSH is a major regulator of thyroid hormone production in the thyroid gland, stimulating the production of thyroxine (T4; 80%) and triiodothyronine (T3; 20%), with T3 being the most active form. Both T3 and T4 exert negative feedback on the hypothalamus and the anterior pituitary, suppressing the secretion of TRH and TSH, respectively, thereby regulating thyroid function and ensuring homeostasis [1].

Thyroid hormones play a crucial role in the development and function of virtually all tissues. Thyroid hormones regulate metabolism, growth, and organ function by altering gene transcription. They increase basal metabolic rate and thermogenesis by upregulating Na⁺/K⁺-ATPase, thereby increasing oxygen consumption and energy expenditure. They enhance carbohydrate and lipid metabolism and stimulate protein synthesis. Cardiovascular effects include increased β-adrenergic receptor expression, resulting in higher heart rate, contractility, and cardiac output. They stimulate respiratory drive, support normal brain development and cognition, promote bone growth and turnover (especially in children), and influence fertility and menstrual function [1].

Physiology in the hospital setting: why thyroid tests become abnormal

The Hypothalamic-Pituitary-Thyroid Axis (HPT) Under Stress

The HPT axis is exquisitely sensitive to systemic stress. Acute illness, inflammatory cytokines, malnutrition, and medications alter thyroid hormone production, peripheral conversion, binding proteins, and receptor activity. These alterations are not necessarily pathological. In many cases, they represent adaptive responses designed to conserve energy during critical illness, predominantly beneficial for survival. The thyroid hormone derangement during acute illness is termed non-thyroidal illness syndrome (NTIS) or euthyroid sick syndrome [8,9].

Non-Thyroidal Illness Syndrome (NTIS)/Euthyroid Sick Syndrome

NTIS refers to transient alterations in thyroid function tests during systemic illness without intrinsic thyroid pathology. Older people are at higher risk because of multiple comorbidities. Although the prevalence of unrecognized thyroid disease among hospitalized patients is 1% to 2.5%, NTIS is observed in up to 60% to 75% of hospitalized patients. Risk factors include infection, stroke, myocardial infarction, liver injury, burns, organ transplantation, malnutrition, malignancy, recent surgery, cardiac or renal failure, and multiple medications [8,10].

The typical biochemical pattern is low total and free T3 (the earliest and most common change), normal or low T4 (in severe illness), and low, normal, or mildly elevated TSH (in the recovery phase). The probability of death correlates with serum total T4 levels. Mortality risk increases as serum total T4 levels decline. When total T4 falls below 4 mcg/dL, the estimated mortality approaches 50%, and when levels drop below 2 mcg/dL, the risk of death exceeds 80% [11]. The proposed mechanisms of NTIS include decreased peripheral conversion of T4 to T3, increased reverse T3, cytokine-induced hypothalamic suppression, decreased deiodinase activity, and altered thyroid-binding globulin levels [10].

NTIS does not require thyroid hormone replacement in most cases. Treatment of the underlying illness typically normalizes laboratory values. The high prevalence of NTIS in hospitalized patients significantly reduces the specificity of TSH testing. Mild TSH abnormalities (0.1-0.6 mIU/L or 6.7-20 mIU/L) have very low positive likelihood ratios for true thyroid disease and may even reduce post-test probability. Only markedly abnormal TSH levels (<0.01 or >20 mIU/L) increase the likelihood of underlying thyroid dysfunction [12]. Large inpatient studies demonstrate that while abnormal TSH values are common, true thyroid disease is uncommon and typically associated with extreme TSH values. Routine admission screening rarely alters management; in one study, only 0.5% of patients experienced a change in clinical care, and these cases had either strong clinical suspicion or known thyroid-related medication use [5].

Subclinical Hypothyroidism

Subclinical hypothyroidism (SCH) is defined as an elevated serum TSH level with a normal FT4 level, and approximately 90% of patients with SCH have a TSH <10 mIU/L. It is often considered an early or mild form of hypothyroidism that may progress to overt disease. An increase in TSH with a normal FT4 is a common phenomenon in the elderly population and obese females. SCH is common in various chronic diseases, such as heart failure, chronic kidney disease, and end-stage renal failure on hemodialysis, unrelated to thyroid disease. However, interpretation in hospitalized patients is complex. During recovery from NTIS, transient elevations in TSH may occur, sometimes reaching up to 20 mIU/L. SCH is often transient, does not require treatment, and requires follow-up of thyroid function in four to six weeks after recovery [13].

Central Hypothyroidism

Central hypothyroidism results from pituitary or hypothalamic dysfunction and is characterized by low or inappropriately normal TSH levels in the presence of low free T4. It is rarely isolated and is more commonly seen in conjunction with other pituitary hormone deficiencies. Compared with primary hypothyroidism, clinical manifestations of central hypothyroidism might be masked by symptoms of hypoadrenalism and hypogonadism. Unlike primary hypothyroidism, TSH may not be elevated in response to low T3 and T4 levels, but may be low, normal, or high, making isolated TSH testing insufficient for diagnosis. So, the diagnosis of central hypothyroidism requires low free T3 and T4 levels together with low or normal TSH concentration. In hospitalized patients, central hypothyroidism may be difficult to distinguish from NTIS, particularly in critically ill patients or those receiving dopamine or glucocorticoids. Clinical suspicion should arise in patients with known pituitary disease, recent pituitary surgery, traumatic brain injury, infiltrative disorders, or concomitant deficiencies in other pituitary hormones. When suspected, evaluation should include assessment of adrenal function before initiating thyroid hormone replacement, and is monitored by serum free T4 levels with the aim of keeping free T4 and T3 levels in the upper half of the normal range [14,15].

Medications affecting thyroid hormones in hospitalized patients

Medications can alter thyroid hormone levels at multiple levels, including the hypothalamus, anterior pituitary gland, thyroid hormone synthesis or release, direct damage to thyroid glands, thyroid hormone metabolism, or by altering the levels or affinity of thyroxine binding globulin (Table 1). Several medications can alter TSH levels without causing true thyroid disease. Dopamine agonists, glucocorticoids, octreotide, and amphetamines can suppress TSH to low but detectable levels, whereas dopamine antagonists such as metoclopramide may cause mild TSH elevation. These changes are typically transient and do not reflect intrinsic thyroid dysfunction. Drugs with high iodine content, such as amiodarone, lithium, and iodinated contrast agents, may affect thyroid hormone release from thyroid glands, causing hypothyroidism. Certain drugs can cause direct damage to thyroid glands, leading to destructive thyroiditis, such as amiodarone or tyrosine kinase inhibitors (sunitinib). Drugs such as amiodarone, dexamethasone, propranolol at high doses, and propylthiouracil inhibit the conversion of T4 to T3, whereas drugs such as phenobarbital, phenytoin, carbamazepine, and rifampin induce glucuronidation enzymes, necessitating an increase in the dose of levothyroxine. Certain medications, including nonsteroidal anti-inflammatory drugs, antiepileptic agents such as phenytoin and carbamazepine, furosemide, and heparin, can displace thyroid hormones from thyroid-binding globulin, leading to transient increases in free T4 and T3 levels with a corresponding decrease in TSH. Drugs such as oral estrogen and selective estrogen-receptor modulators, methadone, heroin, mitotane, and fluorouracil lead to increases in thyroxine-binding globulin, resulting in increased dose requirement of thyroid hormones [16,17]. Medication review is critical before diagnosing thyroid disease.

**Table 1:** Medications affecting thyroid hormones TSH: Thyroid stimulating hormone

Medicine effect	Medicine
TSH suppression	Dopamine agonists, glucocorticoids, octreotide, amphetamines, metformin, synthetic retinoid (bexarotene)
Inhibition of thyroid hormone synthesis and release	Propylthiouracil, methimazole, amiodarone, lithium, iodinated contrast agents
Destructive thyroiditis	Amiodarone, tyrosine kinase inhibitors (sunitinib)
Affecting thyroid hormone metabolism	Amiodarone, dexamethasone, propranolol (at high doses), propylthiouracil, phenobarbital, phenytoin, carbamazepine, rifampin
Altering thyroxine binding globulin	Nonsteroidal anti-inflammatory drugs, antiepileptic agents (phenytoin and carbamazepine), furosemide, heparin, estrogen-containing drugs, methadone, heroin, mitotane, fluorouracil

When should thyroid hormone testing be considered in hospitalized patients?

Routine screening for thyroid hormones in all hospitalized patients is not recommended. The American Association of Clinical Endocrinologists (AACE), the American Thyroid Association (ATA), and the American Thyroid Association Task Force recommend measuring TSH in hospitalized patients only when there is a strong clinical suspicion of thyroid dysfunction [18,19]. In the inpatient setting, abnormal thyroid function tests are frequently due to NTIS and often do not reflect true intrinsic thyroid disease. Therefore, testing should be guided by symptoms, clinical findings, and pretest probability rather than performed routinely.

Thyroid hormone testing is appropriate when patients present with the clinical manifestations (signs or symptoms) suggestive of thyroid disorders [20,21] (Table 2), unexplained cardiovascular instability, new-onset atrial fibrillation, worsening or refractory heart failure, unexplained altered mental status, severe electrolyte abnormalities (e.g., hyponatremia/SIADH), known thyroid disease with decompensation, a known history of medication that affects thyroid function, or concerns for thyroid emergencies [8] (Table 3).

**Table 2:** Signs and symptoms of thyroid disorders

Hyperthyroidism	Hypothyroidism
Weight loss, heat intolerance, sweating, palpitations, tachycardia, atrial fibrillation, widened pulse pressure, anxiety, irritability, tremors, hyperreflexia, insomnia, increased bowel movements, proximal muscle weakness, muscle wasting, oligomenorrhea/amenorrhea, infertility, goiter, ophthalmopathy (Graves’)	Weight gain, cold intolerance, bradycardia, diastolic hypertension, pericardial effusion, fatigue, slowed cognition, depression, hyporeflexia, constipation, muscle cramps, muscle stiffness, dry skin, hair loss, menorrhagia, infertility, hoarseness, macroglossia

**Table 3:** Clinical scenarios where thyroid hormone testing should be considered

Clinical manifestations suggestive of thyroid disorders
Unexplained cardiovascular instability
New-onset atrial fibrillation
Worsening or refractory heart failure
Unexplained altered mental status
Severe electrolyte abnormalities (e.g., hyponatremia/SIADH)
Known thyroid disease with decompensation
History of medications affecting thyroid function
Concerns for thyroid emergencies

Thyroid emergencies in hospitalized patients

Myxedema Coma and Thyroid Storm are the most common thyroid emergencies requiring prompt diagnosis and treatment [22].

Myxedema Coma

Myxedema coma is a rare, life-threatening manifestation of severe, long-standing hypothyroidism characterized by altered mental status and multiorgan decompensation. Despite its name, patients may not be comatose but typically present with confusion, lethargy, or decreased consciousness. It is often precipitated by infection, cold exposure, myocardial infarction, stroke, surgery, or sedative use. Clinical features include hypothermia, bradycardia, hypotension, hypoventilation, hyponatremia, and hypoglycemia. Laboratory findings show markedly elevated TSH with low free T4 in primary hypothyroidism, although central causes may present with low or normal TSH. Mortality remains high, particularly in elderly patients. Myxedema coma is a clinical diagnosis, so management requires immediate ICU care with intravenous levothyroxine, empiric stress-dose glucocorticoids (until adrenal insufficiency is excluded), supportive measures including ventilatory support, cautious fluid management, and treatment of the precipitating cause. Early recognition and prompt treatment are critical to improving outcomes [22].

Thyroid Storm

Thyroid storm is the most extreme manifestation of thyrotoxicosis, characterized by a dramatic clinical picture and multiorgan dysfunction. It typically occurs in patients with untreated or poorly controlled hyperthyroidism and is often precipitated by infection, surgery, trauma, myocardial infarction, or medication nonadherence. Clinical features include high fever, marked tachycardia (often atrial fibrillation), hypertension followed by hypotension, agitation or delirium, tremors, nausea, vomiting, and diarrhea. Laboratory findings show low TSH with elevated free T4 and/or T3, though the degree of hormone elevation does not necessarily correlate with severity. The management of thyroid storm requires immediate ICU care and a multimodal approach: beta-blockers (e.g., propranolol) to control adrenergic symptoms, thionamides (propylthiouracil or methimazole) to inhibit hormone synthesis, iodine (after thionamide administration) to block hormone release, glucocorticoids to reduce peripheral conversion of T4 to T3 and address potential adrenal insufficiency, and treatment of the precipitating trigger. Early recognition and rapid intervention are essential to reduce mortality [22,23].

Interpreting abnormal thyroid function tests

Mild TSH Suppression (0.1-0.4 mIU/L)

Mild TSH suppression is commonly seen in hospitalized patients, especially during acute illness or medication use [3,8]. This change is usually temporary and may indicate non-thyroidal illness syndrome or be caused by medications. If there are no clinical signs of hyperthyroidism, this level of TSH suppression generally does not require treatment. Instead, repeat thyroid function tests should be postponed until recovery from the acute illness, ideally in an outpatient setting, to prevent misdiagnosis and unnecessary treatment.

Mild TSH Elevation (<10 mIU/L)

Mild TSH elevation is often seen during the recovery phase of acute illness and may simply be a temporary adaptive response rather than true hypothyroidism. This is especially common among hospitalized patients and should be interpreted with caution. Starting thyroid hormone replacement therapy in the hospital is generally not advised unless there are obvious clinical reasons or ongoing abnormal results. Follow-up testing after recovery is important to determine whether abnormality persists and whether further evaluation or treatment is needed [13].

Marked Abnormalities

Marked deviations in TSH levels, whether significantly suppressed or elevated, especially when accompanied by corresponding changes in free thyroid hormone levels and consistent clinical features, require prompt evaluation. These findings are more likely to indicate true thyroid dysfunction rather than temporary changes due to illness. The urgency of treatment should be determined by the severity of biochemical abnormalities, the presence of symptoms, and the overall stability of the patient. In such situations, prompt diagnosis and appropriate treatment are essential to prevent complications (Table 4).

**Table 4:** Severity of thyroid function abnormality and recommended action TSH: Thyroid stimulating hormone, NTIS: Non-thyroidal illness syndrome

Pattern of thyroid hormones	Likely Interpretation	Recommended Action
TSH <0.1 + elevated FT4	Overt hyperthyroidism	Initiate therapy; consider endocrinology
TSH >20	Overt hypothyroidism	Treat if clinically appropriate
Low TSH + low FT4	NTIS vs central hypothyroidism	Evaluate the pituitary axis; consider endocrinology

**Figure 1:**
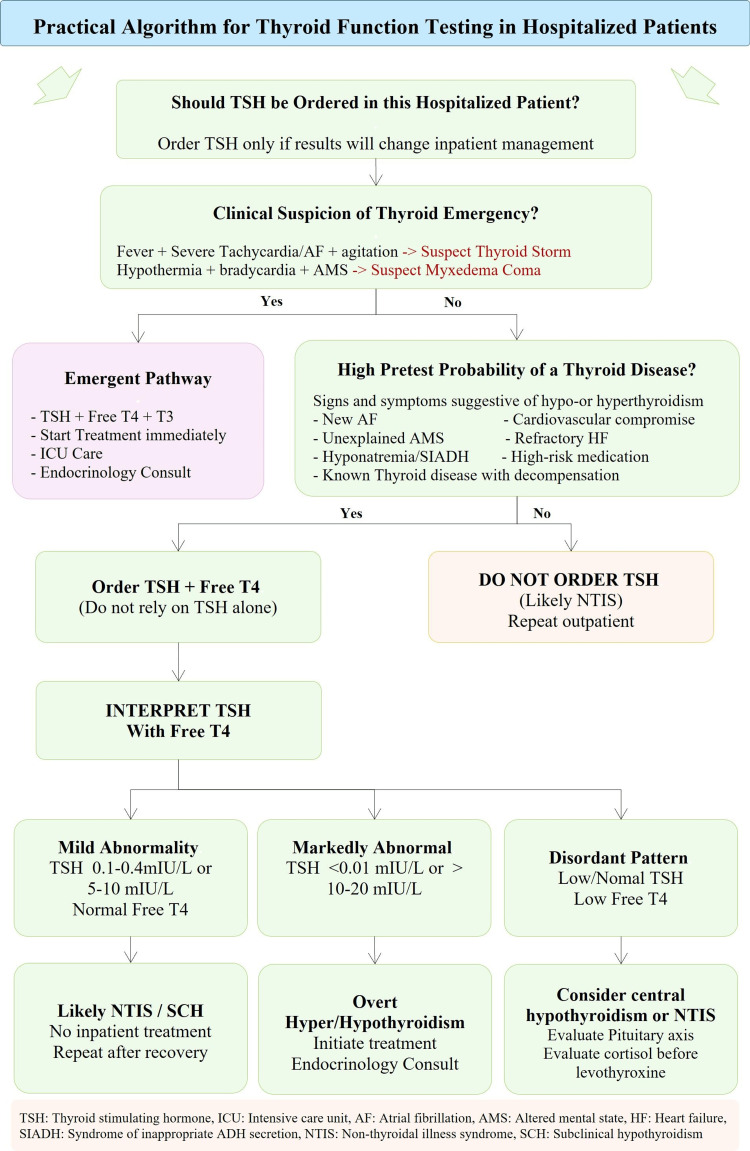
Practical algorithm for thyroid hormone testing in hospitalized patients The image was created by the corresponding author using microsoft excel

Figure 1 shows the proposed practical algorithm for thyroid hormone testing in hospitalized patients in the following steps.

Step 1: Assess the clinical context - Before ordering thyroid function tests, clinicians should evaluate whether thyroid dysfunction is clinically suspected and whether testing will influence immediate management. Key considerations include: suspicion of thyroid emergency, presence of symptoms or signs suggestive of hyper- or hypothyroidism, high pretest probability of thyroid disease, such as new-onset atrial fibrillation, unexplained altered mental status, refractory hyponatremia, unexplained heart failure or persistent tachycardia, and history of thyroid disease. Routine thyroid testing in the absence of clinical suspicion is generally discouraged because thyroid function tests are frequently altered by acute illness.

Step 2: Review medication - several commonly used inpatient medications can alter thyroid function tests or interfere with their interpretation. Clinicians should review the current medication before interpreting results.

Step 3: Interpret TSH with free T4 - the recommended approach is to evaluate TSH along with FT4, not in isolation, with consideration to FT3 in selected cases, particularly when hyperthyroidism is suspected. Recognize that NTIS may cause low T3 levels, low or normal TSH, and variable FT4 levels. Mild abnormalities in TSH (e.g., 0.1-0.4 mIU/L or mildly elevated values) are common during acute illness and often normalize after recovery. When thyroid dysfunction is uncertain, repeat testing after resolution of acute illness is often the most appropriate strategy.

Step 4: Initiate treatment only when clearly indicated - treatment of thyroid dysfunction in hospitalized patients should generally be reserved for situations in which true thyroid disease is evident, such as thyroid emergencies, overt hyper-or hypothyroidism. In the absence of overt thyroid disease, abnormal thyroid test results in the setting of acute illness usually do not require immediate treatment.

When to consult endocrinology in hospitalized patients

Although most inpatient thyroid function test abnormalities are transient and related to non-thyroidal illness syndrome (NTIS), certain clinical scenarios warrant endocrinology consultation. Early involvement of specialists is particularly important when diagnostic uncertainty may alter management, high-risk therapies are being initiated, or life-threatening endocrine emergencies are suspected. Endocrinology consultation is recommended in patients with suspected thyroid emergencies, including thyroid storm or myxedema coma, as well as in cases of hemodynamic instability attributed to thyroid dysfunction. Referral is also warranted in severe thyrotoxicosis, particularly when complicated by atrial fibrillation or heart failure. Additional indications include suspected central hypothyroidism or underlying pituitary pathology, markedly abnormal TSH levels (<0.01 mIU/L or >20 mIU/L), and discordant thyroid function tests, such as low or normal TSH with low free T4. Consultation is further advised in cases of amiodarone-induced thyroid dysfunction and in patients with thyroid abnormalities during pregnancy, where specialized management is required.

## Conclusions

Interpreting thyroid function tests in hospitalized patients requires careful clinical judgment and an understanding of the complex physiological changes during acute illness. NTIS is common in this setting and should not be mistaken for intrinsic thyroid dysfunction, as this could lead to unnecessary tests and improper treatment. Accurate assessment involves combining laboratory findings with the overall clinical picture, including illness severity, medication exposures, and trends in thyroid test results. Trainees and clinicians must stay alert to avoid overdiagnosis, especially in cases with mild TSH abnormalities or conflicting lab results. A structured, evidence-based approach to thyroid test interpretation, focusing on reassessment after recovery, recognizing medication effects, and consulting specialists when needed, can reduce diagnostic errors.
